# ATF4- and CHOP-Dependent Induction of FGF21 through Endoplasmic Reticulum Stress 

**DOI:** 10.1155/2014/807874

**Published:** 2014-05-11

**Authors:** Xiao-shan Wan, Xiang-hong Lu, Ye-cheng Xiao, Yuan Lin, Hong Zhu, Ting Ding, Ying Yang, Yan Huang, Yi Zhang, Yan-Long Liu, Zhu-mei Xu, Jian Xiao, Xiao-kun Li

**Affiliations:** ^1^College of Basic Medical Sciences, Jilin University, Changchun 130021, China; ^2^Translation Medicine Research Center, Lishui People's Hospital, Wenzhou Medical University, Lishui, Zhejiang 323000, China; ^3^Molecular Pharmacology Research Center, Key Laboratory of Biotechnology and Pharmaceutical Engineering, School of Pharmaceutical Science, Wenzhou Medical University, Wenzhou, Zhejiang 325035, China; ^4^Department of Ophthalmology, Xiamen Hospital of Traditional Chinese Medicine, Xiamen 361000, China; ^5^Department of Endocrinology, The First Affiliated Hospital, Wenzhou Medical University, Wenzhou 323000, China

## Abstract

Fibroblast growth factor 21 (FGF21) is an important endogenous regulator involved in the regulation of glucose and lipid metabolism. FGF21 expression is strongly induced in animal and human subjects with metabolic diseases, but little is known about the molecular mechanism. Endoplasmic reticulum (ER) stress plays an essential role in metabolic homeostasis and is observed in numerous pathological processes, including type 2 diabetes, overweight, nonalcoholic fatty liver disease (NAFLD). In this study, we investigate the correlation between the expression of FGF21 and ER stress. We demonstrated that TG-induced ER stress directly regulated the expression and secretion of FGF21 in a dose- and time-dependent manner. FGF21 is the target gene for activating transcription factor 4 (ATF4) and CCAAT enhancer binding protein homologous protein (CHOP). Suppression of CHOP impaired the transcriptional activation of FGF21 by TG-induced ER stress in CHOP−/− mouse primary hepatocytes (MPH), and overexpression of ATF4 and CHOP resulted in FGF21 promoter activation to initiate the transcriptional programme. In mRNA stability assay, we indicated that ER stress increased the half-life of mRNA of FGF21 significantly. In conclusion, FGF21 expression is regulated by ER stress via ATF- and CHOP-dependent transcriptional mechanism and posttranscriptional mechanism, respectively.

## 1. Introduction

The fibroblast growth factor family contains 22 members with a wide range of biological functions relevant to regulating cell growth, differentiation, wound healing, development, and angiogenesis [1–3]. Fibroblast growth factor 21 (FGF21) is a unique member of the FGF family and has broad metabolic functions, including stimulating glucose uptake insulin-independently and improving hyperglycemia and dyslipidemia [4–7]. FGF21 has a protective effect on the preservation of pancreatic *β*-cell function and promotes hepatic and peripheral insulin sensitivity via the prevention of lipolysis, which improves insulin resistance [8–10]. In addition, FGF21 can resist the diet-induced obesity and induce fatty acid oxidation [8, 11, 12]. At present, FGF21 is considered as a novel metabolism regulator and has become a focus of metabolic disease research.

FGF21 is expressed predominantly in liver and, to a lower extent, in white adipose tissue, thymus, skeletal muscle, and pancreatic *β*-cells [4, 9, 13]. Substantial clinical research has focused on detecting FGF21 expression levels in various pathological states. It has been reported that serum FGF21 and hepatic mRNA expression levels in patients with NAFLD are significantly higher than levels in control subjects, which correlates with a substantial increase in liver triglyceride levels [14–16]. Plasma FGF21 was also found to elevate in type 2 diabetic or impaired glucose tolerance patients [17–19]. Circulating FGF21 levels were significantly higher in overweight subjects than those in lean individuals [20, 21]. Animal studies have reported similar results, showing increased FGF21 mRNA levels and serum FGF21 concentrations in the hepatic and adipose tissue of high fat diet-induced and genetically obese mice compared with wild-type mice [6, 8, 22]. An increase in FGF21 mRNA levels is similarly induced by fasting [23–25]. It seems likely that FGF21 levels are unchanged in different physiological states but increased with stress in individuals who are either overweight or have type 2 diabetes, or NAFLD. Based on these findings, we propose that the mechanism of increased FGF21 levels in metabolism disease may be due to feedback regulation, but the mechanism responsible for the effect is still unclear.

Numerous studies indicated that ER stress was closely related to metabolic diseases and it contributed to triggering insulin resistance, obesity, and type 2 diabetes [26–29]. ER is the site of synthesis, folding, and routing of proteins and it plays a prominent role in maintaining Ca^2+^ homeostasis in the cytosol. ER stress is a compensatory process that aims to preserve cellular functions and survival and induce by hypoxia, toxicity, infection, unfold protein accumulation, and perturbation of Ca^2+^ homeostasis [30]. ER stress transducers, including PKR-like ER kinase (PERK), activating transcription factor 6 (ATF6), and inositol-requiring enzyme 1 (IRE1), can be activated [31]. Phosphorylation of eukaryotic initiation factor *α* (eIF2*α*), via activation by PERK, leads to translational induction of ATF4. BiP-free pATF6(p) is transported to the Golgi apparatus where it is processed to a transcriptionally active nuclear form pATF6(N). Activated IRE1 site-specifically cleaves x-box-binding protein 1 (XBP1) mRNA precursor to create the mature XBP1 mRNA (XBP1-sp). ATF4, pATF6(N), and XBP1-sp then activate transcription of CCAAT enhancer binding protein homologous protein (CHOP) by binding to the appropriate promoter region, and CHOP plays a crucial role in ER stress-mediated apoptosis and in diseases including diabetes, brain ischemia, and neurodegenerative disease [32].

Several studies have shown that upregulation of FGF21 is mediated by ATF4 under conditions causing cellular stress, such as amino acid deprivation, autophagy, and mitochondrial dysfunction [33–36]. ATF4 directly increases FGF21 expression in cells with ER stress by binding to both amino acid-responsive element 1 (AARE1) and amino acid-responsive element 2 (AARE2) sequence on FGF21 [35, 37]. ATF4 activates the CHOP gene downstream, but not much is known on the relationship between CHOP and FGF21. To investigate whether FGF21 is regulated by ER stress via effects on AFT4 and CHOP, we establish an ER stress cell model using TG (thapsigargin) in which we detect FGF21 and ER stress-specific gene expression levels. We then demonstrated that TG-induced ER stress upregulates the expression and secretion of FGF21 by influencing ATF4 and CHOP, providing insights on the mechanisms that link FGF21 and metabolic diseases.

## 2. Materials

Dulbecco's modified Eagle's medium (DMEM), penicillin-streptomycin (p-s), newborn calf serum (NCS), and fetal bovine serum (FBS) were obtained from Gibco BRL (Grand Island, NY, USA). TRIzol reagent was obtained from Invitrogen (Carlsbad, CA, USA). High-Capacity cDNA Reverse Transcription Kits were obtained from Applied Biosystems (Foster City, CA, USA). QIAprep spin miniprep kits were obtained from Qiagen. Restriction endonucleases* Hind* III and* Xho* I were purchased from NEB (Ipswich, MA, USA). Vector pGL4.17-Luc, Fugene HD reagents, and Luciferase Assay System were obtained from Promega (Sunnyvale, CA, USA). Mouse FGF21 ELISA Kits were obtained from R&D Systems (Minneapolis, MN, USA). Isobutyl-1-methylxanthine (IBMX), Dexamethasone (DEX), Insulin, Thapsigargin (TG), Actinomycin D, and all other chemical reagents were obtained from Sigma-Aldrich (St. Louis, MO).

## 3. Methods

### 3.1. Cell Culture and Differentiation

3T3-L1 murine preadipocytes were obtained from the American Type Culture Collection (Manassas, VA, USA). Cells were cultured in DMEM containing 10% NCS and 1% p-s; cells were induced to differentiate with DMEM plus 10% FBS, 1% p-s, 0.5 mM IBMX, 1 *μ*M of DEX, and 1.7 *μ*M Insulin for two days. Then the induction medium was replaced by DMEM with 10% FBS, 1% p-s, and 1.7 *μ*M Insulin for another two days, followed by 10% FBS/DMEM medium, which was changed every two days. After 5-6 additional days, more than 85% cells differentiated to mature adipocytes, which can be used for the experiments.

### 3.2. Isolation and Culture of Mouse Primary Hepatocytes

Primary hepatocytes were isolated from C57BL/6J wild type (WT) and CHOP knockout (CHOP−/−) mice (male, 8 weeks) and cultured as described previously [38]. Cells were maintained in serum-free William'E medium containing 0.1 *μ*M Dex, 1% penicillin, and 1 *μ*M thyroxine. Before treatment, cells were incubated at 37°C, in 5% CO_2_ for approximately 16 h or until they had attached.

### 3.3. RNA Isolation and Real-Time Reverse Transcription-Polymerase Chain Reaction (RT-PCR)

Total RNA was extracted from 3T3-L1 adipocytes using the TRIzol reagent according to the manufacturer's instructions. Total RNA (2 *μ*g) was used as a template for first-strand cDNA synthesis using the High-Capacity cDNA Reverse Transcription Kit. The mRNA levels of ATF4, splicing of XBP1 (XBP1-sp), CHOP, and FGF21 were quantified using the following primers. ATF4 forward primer 5′-CCT AGG TCT CTT AGA TGA CTA TCT GGA GG-3′, ATF4 reverse primer 5′-CCA GGT CAT CCA TTC GAA ACA GAG CAT CG-3′; XBP1-sp forward primer 5′-TGA GTC CGC AGC AGG TG-3′, XBP1-sp reverse primer 5′-GAC AGG GTC CAA CTT GT-3′; CHOP forward primer 5′-GCT CCT GCC TTT CAC CTT GG-3′, CHOP reverse primer 5′-GGT TTT TGA TTC TTC CTC TTC-3′; FGF21 forward primer 5′-GCA GTC CAG AAA GTC TCC-3′, FGF21 reverse primer 5′-TGT AAC CGT CCT CCA GCA G-3′; iQ SYBR Green Supermix was used as a fluorescent dye to detect the presence of double-stranded DNA. The mRNA levels of each target gene were normalized to an endogenous control Glyceraldehyde-3-phosphate dehydrogenase (GAPDH). GAPDH forward primer 5′-GTC GTG GAT CTG ACG TGC C-3′, GAPDH reverse primer 5′-GAT GCC TGC TTC ACC ACC TT-3′. The ratio of normalized mean value for each treatment group to vehicle control group (DMSO) was calculated.

### 3.4. Enzyme-Linked Immunosorbent Assay (ELISA) of FGF21

3T3-L1 adipocytes were treated with TG (0, 12.5, 25, 50, and 100 nM) for 24 h, or TG (100 nM) for 0, 2, 4, 8, 16, and 24 h. The accumulated FGF21 in the culture medium was determined using ELISA Kit according to the manufacturer's instructions. The total protein concentrations of viable cells were determined using the Bio-Rad Protein Assay reagent. The total amounts of the FGF21 in medium were normalized to the total protein amounts and reported as pg/mg protein.

### 3.5. Plasmids Construction and Luciferase Assay

The mouse FGF21 promoter constructs −1497/+5 were generously provided by Dr. Wenke Feng (The University of Louisville, Louisville, USA) and subcloned into pGL4.17-Luc luciferase report vector using* Hind* III and* Xho* I sites. The expression vector containing the coding sequence of ATF4 or CHOP was preserved in our laboratory. All plasmids were propagated in* Escherichia coli* DH5*α* and isolated using QIAprep spin miniprep kit (Qiagen). 293T cells were plated in 6-well plates 24 h before transfection. Cells were transfected with 2 *μ*g of pGL4.17 promoter FGF21 (−1497/+5), 2 *μ*g of ATF4, or CHOP expression vector using Fugene HD (Promega). 48 h after transfection, the cells were harvested and lysed, and the luciferase activity was measured using the Luciferase Assay System (Promega). The transfection efficiency was normalized to cotransfection of 1 *μ*g of GFP vector.

### 3.6. Assessment of FGF21 mRNA Stability

3T3-L1 mature adipocytes were treated with TG (100 nM)/DTT or vehicle control for 4 h; then Actinomycin D (5.0 *μ*g/mL) was added to the medium (time 0 h). The mRNA of the cells was isolated after added Actinomycin D for 0.5, 1, 2, 4, and 6 h. FGF21, ATF4, XBP1-sp, and CHOP mRNA levels were detected using real-time RT-PCR as described in the previous section; the results are expressed as the fold of the mRNA value at the time of Actinomycin D addition.

### 3.7. Statistical Analysis

All of the experiments were repeated at least three times; results were stated as the mean ± standard error. One-way ANOVA was employed to analyze the differences between sets of data. Statistics were performed using GraphPad Pro. A value of *P* < 0.05 was considered significant.

## 4. Results

### 4.1. ER Stress Increases FGF21 Expression

**Figure 1:**
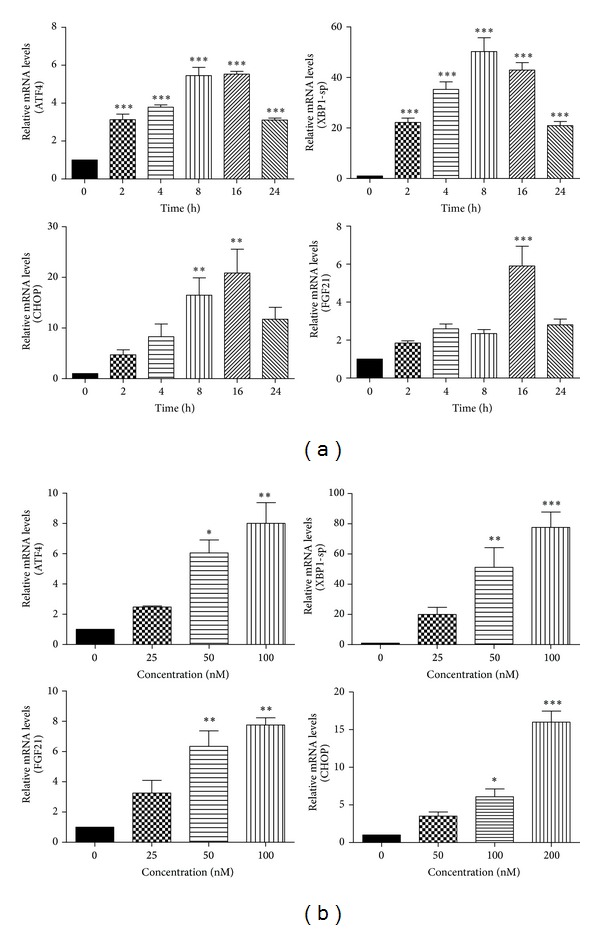
ER stress increases FGF21 mRNA levels. (a) 3T3-L1 adipocytes were treated with TG (100 nM) for 0, 2, 4, 8, 16, and 24 h; (b) 3T3-L1 adipocytes were treated with TG (25, 50, and 100 nM) for 16 h. Total cellular RNA was isolated. The mRNA levels of ATF4, XBP1-sp, CHOP, and FGF21 were measured by real-time RT-PCR. Values are mean ± S.E. of three independent experiments. Statistical significance relative to vehicle control: *P < 0.05; **P < 0.01; ***P < 0.001.

To investigate the effect of ER stress on FGF21 mRNA levels, we treated 3T3-L1 adipocytes with TG, a potent ER stress activator, by disturbing ER calcium homeostasis. The mRNA levels of ER stress-specific genes (ATF4, XBP1-sp, and CHOP) and FGF21 were detected using real-time RT-PCR. We observed that TG increased FGF21mRNA expression in a time-dependent manner (Figure 1(a)). However, the expression levels at 24 h were lower than those at 16 h, perhaps due to cell toxicity. As shown in Figure 1(b), after the 3T3-L1 adipocytes were incubated with 12.5, 25, and 100 nM TG for 16 h, the levels of FGF21 mRNA were significantly increased in a concentration-dependent manner compared with the vehicle control group.

### 4.2. ER Stress Induces FGF21 Secretion

**Figure 2:**
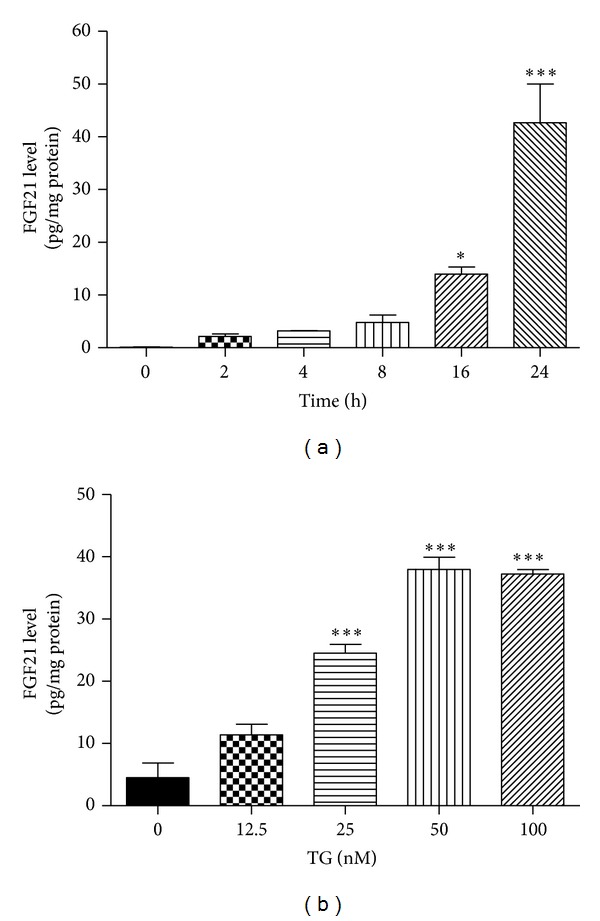
ER stress induces FGF21 secretion. Differentiated 3T3-L1 cells were treated with 100 nM TG for 0, 2, 4, 8, 16, and 24 h (a); or different concentrations of TG for 24 h (b), at the end of treatment, cell culture medium was collected. The protein level of FGF21 was determined by ELISA. Values are mean ± S.E. of three independent experiments. Statistical significance relative to vehicle control: *P < 0.05; ***P < 0.001.

Based on the above findings, a model of TG-induced stress in 3T3-L1 adipocytes was established, and we used this model to examine whether ER stress increases FGF21 secretion. Differentiated 3T3-L1 cells were treated with TG; the FGF21 protein levels in the medium were measured using ELISA. As shown in Figures 2(a) and 2(b), TG-induced ER stress led to increase in secreted FGF21 in a time- and dose-dependent manner. TG induced FGF21 protein level to a 40-fold rise at concentration of 100 nM for 24 h.

### 4.3. Knockout of CHOP Decreases FGF21 Expression

**Figure 3:**
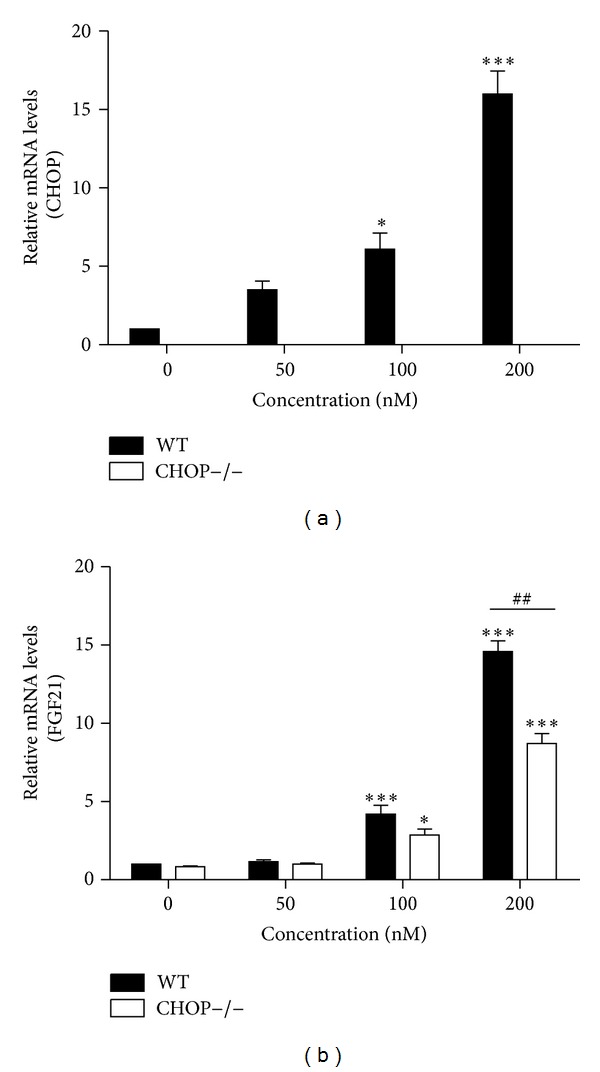
Knockout of CHOP decreases FGF21 expression. WT and CHOP−/− mouse primary hepatocytes were treated for 24 h with increasing concentration of TG. Total cellular RNA was isolated and the mRNA levels of CHOP and FGF21 were measured by real-time RT-PCR. Values are mean ± S.E. of three independent experiments. Statistical significance relative to WT vehicle control: *P < 0.05; ***P < 0.001; statistical significance relative of the same TG concentration between WT group and CHOP−/− group: ^##^ *P* < 0.01.

CHOP is a major transcription factor involved in ER stress. To determine whether CHOP expression contributes to ER stress-induced upregulation of FGF21, we isolated MPH from WT and CHOP−/− mice and treated the cells with TG for 24 h. In WT MPH, TG promoted the mRNA levels of CHOP and FGF21. However, in CHOP−/− MPH, TG failed to induce FGF21 expression, because ATF4 is upstream gene of CHOP and there is no effect of CHOP knockout on the activation of ATF4. Moreover, much research indicated that ATF4 can induce FGF21 expression under stress [33–37]. As depicted in Figure 3, an absence of CHOP expression significantly increased FGF21 gene expression by 30%. These results indicate that CHOP may be a key player in the mechanism by which TG-induces increased FGF21 expression in MPH.

### 4.4. ATF4 and CHOP Increase FGF21 Promoter-Driven Transcription

**Figure 4:**
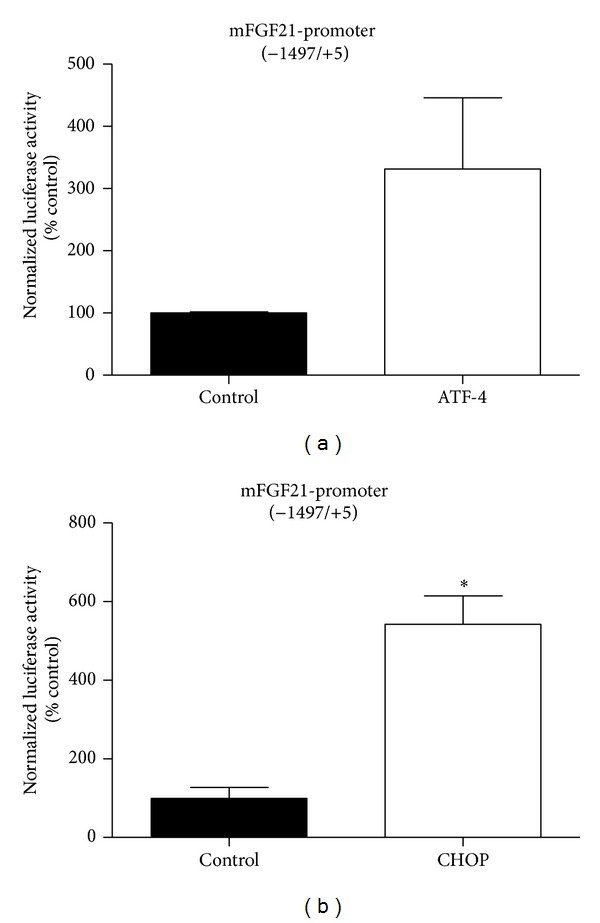
ATF4 and CHOP increase FGF21 promoter-driven transcription. 293T cells were transfected with FGF21 promoter reporter construct along with the expression plasmid ATF4 or CHOP. Values are mean ± S.E. of three independent experiments. Statistical significance relative to control vector: *P < 0.05.

To address the mechanism of TG-induced stress regulating FGF21 expression, we subcloned the FGF21 promoter (−1497/+5) into the pGL4.17-Luc luciferase report vector and measured the ability of ATF4 or CHOP to regulate the activation of the FGF21 promoter using a cotransfection assay. A previous study has reported that FGF21 expression could be mimicked by overexpression of ATF4 [37]. Unlike 3T3-L1 cell line, 293T cells can be transfected with high efficiency. 293T cells were cotransfected with pGL4.17-Luc luciferase report vector, which was inserted the mouse FGF21 promoter and expression vector for ATF4 or CHOP. Luciferase activity was determined at 48 h after transfection.  Figure 4(a) demonstrates that, compared with the control group, ATF4 overexpression enhanced FGF21 promoter activity more than 3-fold. This is consistent with the result of a previous study [37] that reported that there are two conserved ATF4-binding sites in the promoter region of the FGF21 gene and that FGF21 expression can be mimicked by overexpression of ATF4. CHOP is one of the genes that is downstream of ATF4. We hypothesized that CHOP would induce FGF21 expression similar to ATF4. We transfected HEK293 cells with the FGF21 reporter construct and CHOP. As shown in Figure 4(b), similar to ATF4, CHOP overexpression significantly increased the transcription of an FGF21 promoter-driven reporter. These findings indicate that ATF4 and CHOP upregulate FGF21 expression by activating the promoter in an environment of TG-induced ER stress.

### 4.5. ER Stress Increases FGF21 mRNA Stability

**Figure 5:**
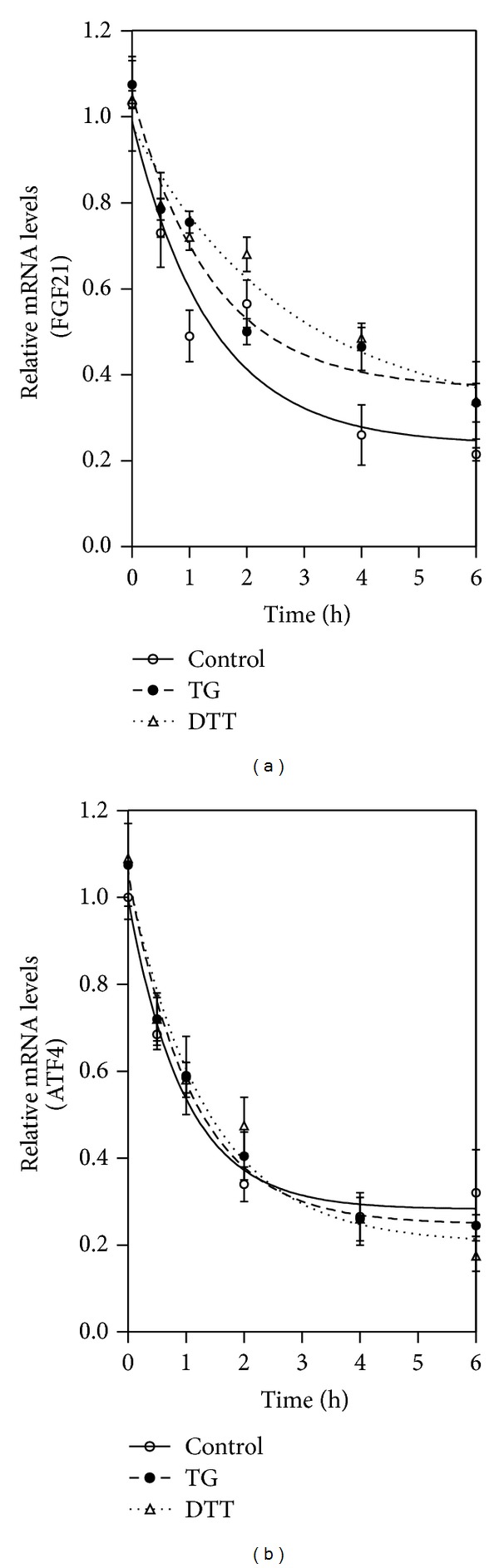
ER stress increases FGF21 mRNA stability. 3T3-L1 adipocytes were pretreated with 100 nM TG/DTT or vehicle control (DMSO) for 4 h and then treated with 5.0 *μ*g/mL actinomycin D (time 0). Total cellular RNA was extracted at 0, 0.5, 1, 2, 4, and 6 h after actinomycin D addition. FGF21 mRNA levels were determined by real-time RT-PCR. Values are mean ± S.E. of three independent experiments.

Posttranscriptional regulation is a major mechanism for the expression of cytokines. To determine whether TG- or DTT- (dithiothreitol-) induced ER stress increases FGF21 expression by regulating mRNA stability, we examined the effects of TG/DTT on the mRNA stability of FGF21 in 3T3-L1 adipocytes. The results indicated that TG and DTT increased the half-life of mRNA of FGF21 significantly but had no effect on ER stress-specific genes (Figure 5). This result suggested TG- and DTT-induced ER stress activate FGF21 expression by increasing mRNA stability specifically.

## 5. Discussion

FGF21 acts as a hormone-like cytokine on multiple tissues to coordinate carbohydrate and lipid metabolism [4]. Clinical research has shown that serum FGF21 levels are higher in subjects who are overweight, have NAFLD, or are type 2 diabetic [14–18, 20]. Similarly, circulating FGF21 concentrations in* db/db* mice were much higher than normal, as were the FGF21 mRNA levels in both the liver and white adipose tissue [6, 8, 22]. Previous studies have reported that FGF21 expression is mediated by several transcriptional activators and their DNA response elements. Gene expression of FGF21 is induced directly by PPAR*α* in response to starvation and ketotic states and PPAR*α* agonists in liver [23, 25] as well as in cultured adipocytes and adipose tissue by PPAR*γ* [39–41]. Activation of the farnesoid X receptor (FXR) increased FGF21 gene expression and secretion was mediated by FXR/retinoid X receptor binding site in 5′-flanking region of the FGF21 gene [42]. A study demonstrated that glucose activation of carbohydrate response element binding protein (ChREBP) is involved in the upregulation of FGF21 mRNA expression in liver [43]. Retinoic acid receptor-related receptor *α* (ROR*α*) also induces expression and secretion of FGF21, and there is a canonical ROR response element in the proximal promoter of FGF21 gene that exhibits functional activity [44]. PGC-1*α*-mediated reduction of FGF21 expression is dependent on the expression of its ligand, ALAS-1, and Rev-Erb*α* [45].

In addition, studies by Schaap et al. suggest that FGF21 expression is regulated by ER stress [37]. The authors reported that FGF21 mRNA is increased by TG-induced ER stress in rat H4IIE cells and rat primary hepatocytes. Moreover, intraperitoneal injection of the ER stressor tunicamycin induced hepatic FGF21 expression in mice and resulted in marked elevation of serum FGF21 levels [37]. Consistent with these new findings, we observed that TG-induced ER stress elevated FGF21 expression and secretion in murine 3T3-L1 adipocytes along with increasing ATF4 expression.

PERK (PKR-like ER kinase) is one of the major ER stress pathways. PERK can induce CHOP via activating ATF4. However, there was no information regarding the regulation of FGF21 by CHOP. We show for the first time that CHOP can increase FGF21 expression by activating transcription via promoter elements and enhancing mRNA stability in ER stress. We analyzed mouse FGF21 (−1497/+5) promoter and confirmed the absence of the conserved CHOP binding site 5′-(A/G) (A/G) TGCAAT (A/C) CCC-3′. Thus, FGF21 was not directly responsive to CHOP directly. To the contrary, our data demonstrates that CHOP can induce the transcription of a FGF21 promoter-driven reporter (Figure 4(b)). CHOP may also regulate the expression of FGF21 indirectly by activating other cytokines and intracellular stress signaling pathways, though this remains to be determined conclusively.

Gene expression can be regulated by posttranscriptional control of mRNA stability [46]. The presence of AU-rich elements (AREs) in the 3′-untranslated region (3′-UTR) is essential for stabilization or degradation of mRNA of inflammatory factor [47]. The RNA-binding proteins (RBPs), such as HuR, AUF1, and CUG-BP1, positively regulate stability of many target mRNA via binding AREs present in the 3′-UTR [48, 49]. In this study, we identified for the first time that increased FGF21 mRNA stability, through the binding of RBPs to its target mRNAs, is responsible for elevated FGF21 levels by TG- or DTT-induced ER stress in differentiated 3T3-L1 cells.

In conclusion, these findings suggest that FGF21 is the target gene for ATF4 and CHOP, and transcription and mRNA stabilization are responsible for ATF4 and CHOP mediated induction of FGF21 expression in ER stress. Thus, we indicate ER stress is the key mechanism for regulating FGF21 in several metabolic diseases. Moreover, our studies provide important information about the FGF21 signaling pathway and the clinical significance of FGF21 in the development of metabolic diseases. Compared with WT MPH, FGF21 mRNA levels are reduced in CHOP−/− MPH treated with TG; however, the effects of CHOP overexpression on FGF21 levels are not understood. And it remains to be detected that the synergistic effect of ATF4 and CHOP on FGF21 expression. Moreover, further prospective studies are needed to determine the specific RBPs and their binding sites in FGF21 3′-UTR as well as the signaling pathway of CHOP- dependent activation of FGF21 in ER stress.
