# Corrigendum to “ATF4- and CHOP-Dependent Induction of FGF21 through Endoplasmic Reticulum Stress”

**DOI:** 10.1155/2018/3218606

**Published:** 2018-05-06

**Authors:** Xiao-shan Wan, Xuan Wang, Jian Xiao, Xiao-kun Li, Huiping Zhou

**Affiliations:** ^1^College of Basic Medical Sciences, Jilin University, Changchun 130021, China; ^2^Department of Microbiology and Immunology, Medical College of Virginia Campus, McGuire Veterans Affairs Medical Center, Virginia Commonwealth University, Richmond, VA 23298, USA; ^3^Translation Medicine Research Center, Lishui People's Hospital, Wenzhou Medical University, Lishui, Zhejiang 323000, China; ^4^Molecular Pharmacology Research Center, Key Laboratory of Biotechnology and Pharmaceutical Engineering, School of Pharmaceutical Science, Wenzhou Medical University, Wenzhou, Zhejiang 325035, China

In the article titled “ATF4- and CHOP-Dependent Induction of FGF21 through Endoplasmic Reticulum Stress” [1], there were errors that have been corrected in the revised version shown below.

- The article was submitted without the permission of Huiping Zhou, the director of the laboratory.
- Drs. Xuan Wang and Huiping Zhou were missing from the authors' list, and Drs. Xiang-hong Lu, Ye-cheng Xiao, Yuan Lin, Hong Zhu, Ting Ding, Ying Yang, Yan Huang, Yi Zhang, Yan-Long Liu, and Zhu-mei Xu were incorrectly included as authors. The corrected authors' list and affiliations are shown above.
- The acknowledgments were updated.
- The Methods section was revised and now includes detailed information relating to animal protocols and primary cell isolation.

The corrected article is as follows. 

## Abstract

Fibroblast growth factor 21 (FGF21) is an important endogenous regulator involved in the regulation of glucose and lipid metabolism. FGF21 expression is strongly induced in animal and human subjects with metabolic diseases, but little is known about the molecular mechanism. Endoplasmic reticulum (ER) plays an essential role in maintenance of metabolic homeostasis and stress-induced ER dysfunction is involved in numerous pathological processes, including type 2 diabetes, obesity, and nonalcoholic fatty liver disease (NAFLD). In this study, we investigated the correlation between the expression of FGF21 and ER stress. We demonstrate that a well-known ER stress inducer, thapsigargin (TG), directly regulates the expression and secretion of FGF21 in a dose- and time-dependent manner. We further identify that FGF21 is the target gene of the activating transcription factor 4 (ATF4) and CCAAT enhancer binding protein homologous protein (CHOP). TG-induced transcriptional activation of FGF21 is inhibited in mouse primary hepatocytes isolated from CHOP knockout mice. In addition, overexpression of ATF4 and CHOP increases FGF21 promoter activities. Furthermore, activation of ER stress significantly increases the mRNA half-life of FGF21. In summary, hepatic FGF21 expression is regulated by ATF4 and CHOP via both transcriptional mechanisms and posttranscriptional mechanisms.

## 1. Introduction

The fibroblast growth factor family contains 22 members with a wide range of biological functions relevant to regulating cell growth, differentiation, wound healing, development, and angiogenesis [1–3]. Fibroblast growth factor 21 (FGF21) is a unique member of the FGF family and has broad metabolic functions, including stimulating glucose uptake independent of insulin and improving hyperglycemia and dyslipidemia [4–7]. FGF21 has a protective effect on the preservation of pancreatic *β*-cell function and promotes hepatic and peripheral insulin sensitivity via the prevention of lipolysis, which improves insulin resistance [8–10]. In addition, FGF21 can prevent diet-induced obesity and induce fatty acid oxidation [8, 11, 12]. At present, FGF21 is considered as a novel metabolism regulator and has become a focus of metabolic disease research.

FGF21 is expressed predominantly in liver and, to a less extent, in white adipose tissue, thymus, skeletal muscle, and pancreatic *β*-cells [4, 9, 13]. Substantial clinical research has focused on detecting FGF21 expression levels in various pathological states. It has been reported that serum FGF21 and hepatic mRNA expression levels in patients with NAFLD are significantly higher than those in control subjects, which correlates with a substantial increase in hepatic triglyceride levels [14–16]. Plasma FGF21 is also elevated in type 2 diabetic or impaired glucose tolerance patients [17–19]. Circulating FGF21 levels are significantly higher in overweight subjects than in lean individuals [20, 21]. Animal studies also showed that serum FGF21 concentrations and FGF21 mRNA levels in the liver and adipose tissue of high fat diet-induced and genetically modified obese mice are higher compared with those in wild-type mice [6, 8, 22]. In addition, FGF21 mRNA levels are also induced by fasting [23–25]. Under different normal physiological conditions, FGF21 levels remain unchanged. However, under various stress conditions, FGF21 is increased, for example, in individuals who either are overweight or have type 2 diabetes, or NAFLD. These observations suggest that the increased FGF21 levels in metabolic diseases may be due to feedback regulation. However, the underlying mechanism is still unclear.

Numerous studies indicate that ER stress is closely related to metabolic diseases and it contributes to insulin resistance, obesity, and type 2 diabetes [26–29]. ER is the site of synthesis, folding, and routing of proteins and it plays a prominent role in maintaining Ca^2+^ homeostasis in the cytosol. ER stress is a compensatory process that aims to preserve cellular functions and survival and is induced by hypoxia, infection, overloading of unfolded or misfolded proteins, and perturbation of Ca^2+^ homeostasis [30]. Three ER stress transducers, including PKR-like ER kinase (PERK), activating transcription factor 6 (ATF6), and inositol-requiring enzyme 1 (IRE1), have been identified [31]. PERK-mediated phosphorylation of eukaryotic initiation factor *α* (eIF2*α*) leads to termination of protein translation and further induction of downstream transcriptional factor ATF4. BiP-free pATF6(p) is transported to the Golgi apparatus where it is processed to a transcriptionally active nuclear form pATF6(N). Activated IRE1 site-specifically cleaves x-box-binding protein 1 (XBP1) mRNA precursor to create the mature XBP1 mRNA (XBP1-sp). ATF4, pATF6(N), and XBP1-sp can further activate the transcription of numerous genes involved in cell growth and apoptosis including CHOP, which plays a crucial role in ER stress-mediated apoptosis and has been implicated in various human diseases including metabolic diseases, diabetes, various liver diseases, brain ischemia, and neurodegenerative disease [32].

Several studies have shown that upregulation of FGF21 is mediated by ATF4 under cellular stress conditions, such as amino acid deprivation, autophagy, and mitochondrial dysfunction [33–36]. ATF4 directly increases FGF21 expression in cells with ER stress by binding to both of the amino acid-responsive element 1 (AARE1) and amino acid-responsive element 2 (AARE2) sequences on FGF21 [35, 37]. CHOP is downstream of transcriptional factor of ATF4, but little is known regarding the relationship between CHOP and FGF21. To investigate whether ER stress-mediated FGF21 expression is regulated by AFT4 and CHOP, we established an ER stress cell model using thapsigargin (TG), a well-known ER stress inducer. Both FGF21 and ER stress-specific gene expression levels were markedly induced by TG. We further demonstrated that TG-induced ER stress upregulated the expression and secretion of FGF21 via induction of both ATF4 and CHOP. Our findings in this current study provide novel insights in understanding the role of FGF21 in metabolic diseases.

## 2. Materials

Dulbecco's modified Eagle's medium (DMEM), penicillin-streptomycin (p-s), newborn calf serum (NCS), and fetal bovine serum (FBS) were obtained from Gibco BRL (Grand Island, NY, USA). TRIzol reagent was obtained from Invitrogen (Carlsbad, CA, USA). High-Capacity cDNA Reverse Transcription Kits were obtained from Applied Biosystems (Foster City, CA, USA). QIAprep spin miniprep kits were obtained from Qiagen. Restriction endonucleases* Hind* III and* Xho* I were purchased from NEB (Ipswich, MA, USA). Vector pGL4.17-Luc, Fugene HD reagents, and Luciferase Assay System were obtained from Promega (Sunnyvale, CA, USA). Mouse FGF21 ELISA Kits were obtained from R&D Systems (Minneapolis, MN, USA). Isobutyl-1-methylxanthine (IBMX), dexamethasone (DEX), insulin, thapsigargin (TG), actinomycin D, and all other chemical reagents were obtained from Sigma-Aldrich (St. Louis, MO, USA).

## 3. Methods

### 3.1. Cell Culture and Differentiation

3T3-L1 murine preadipocytes were obtained from the American Type Culture Collection (Manassas, VA, USA). Cells were cultured in DMEM containing 10% NCS and 1% p-s. Cells were induced to differentiate with DMEM plus 10% FBS, 1% p-s, 0.5 mM IBMX, 1 *μ*M of DEX, and 1.7 *μ*M insulin for two days. Then the induction medium was replaced by DMEM with 10% FBS, 1% p-s, and 1.7 *μ*M insulin for another two days, followed by 10% FBS/DMEM medium, which was changed every two days. After 5-6 additional days, more than 85% cells differentiated to mature adipocytes, which could be used in the experiments.

### 3.2. Isolation and Culture of Mouse Primary Hepatocytes

C57BL/6J wild-type (WT) and CHOP^−/−^ mice were purchased from Jackson Laboratory (Bar Harbor, ME) and housed under 12 h light/12 h dark cycle with free access to water and normal chow. All animal study protocols were approved by the Institutional Animal Care and Use Committee of Virginia Commonwealth University. Primary hepatocytes were isolated from C57BL/6J WT and CHOP^−/−^ mice (male, 8 weeks) and cultured as described previously [38]. Cells were maintained in serum-free William's E medium containing 0.1 *μ*M DEX, 1% penicillin and 1 *μ*M thyroxine. Before treatment, cells were incubated at 37°C, in 5% CO_2_ for approximately 16 h to be attached. TG was dissolved in DMSO and was added to the culture medium (final concentrations 50–200 nM) and incubated for 24 h.

### 3.3. RNA Isolation and Real-Time Reverse Transcription-Polymerase Chain Reaction (RT-PCR)

Total RNA was extracted from 3T3-L1 adipocytes using the TRIzol reagent according to the manufacturer's instructions. Total RNA (2 *μ*g) was used as a template for first-strand cDNA synthesis using the High-Capacity cDNA Reverse Transcription Kit. The mRNA levels of ATF4, splicing of XBP1 (XBP1-sp), CHOP, and FGF21 were quantified using the following primers: ATF4 forward primer 5′-CCT AGG TCT CTT AGA TGA CTA TCT GGA GG-3′, ATF4 reverse primer 5′-CCA GGT CAT CCA TTC GAA ACA GAG CAT CG-3′; XBP1-sp forward primer 5′-TGA GTC CGC AGC AGG TG-3′, XBP1-sp reverse primer 5′-GAC AGG GTC CAA CTT GT-3′; CHOP forward primer 5′-GCT CCT GCC TTT CAC CTT GG-3′, CHOP reverse primer 5′-GGT TTT TGA TTC TTC CTC TTC-3′; FGF21 forward primer 5′-GCA GTC CAG AAA GTC TCC-3′, FGF21 reverse primer 5′-TGT AAC CGT CCT CCA GCA G-3′. iQ™ SYBR Green Supermix was used as a fluorescent dye to detect the presence of double-stranded DNA. The mRNA levels of each target gene were normalized to the endogenous control glyceraldehyde-3-phosphate dehydrogenase (GAPDH). GAPDH forward primer was 5′-GTCGTGGATCTGACGTGCC-3′. GAPDH reverse primer was 5′-GATGCCTGCTTCACACCTT-3′. The ratio of normalized mean value for each treatment group to vehicle control group (DMSO) was calculated.

### 3.4. Enzyme-Linked Immunosorbent Assay (ELISA) of FGF21

3T3-L1 adipocytes were treated with TG (0, 12.5, 25, 50, and 100 nM) for 24 h, or TG (100 nM) for 0, 2, 4, 8, 16, and 24 h. The accumulated FGF21 in the culture medium was determined using the ELISA Kit according to the manufacturer's instructions. The total protein concentrations of viable cells were determined using the Bio-Rad Protein Assay reagent. The total amounts of the FGF21 in medium were normalized to the total protein amounts and reported as pg/mg protein.

### 3.5. Plasmid Construction and Luciferase Assay

The mouse FGF21 promoter constructs −1497/+5 were generously provided by Dr. Wenke Feng (the University of Louisville, Louisville, USA) and subcloned into pGL4.17-Luc luciferase report vector using* Hind* III and* Xho* I sites. The recombinant expression vectors of ATF4 and CHOP were constructed by subcloning of the coding sequence of ATF4 and CHOP into pcDNA6 vectors. All plasmids were propagated in* Escherichia coli* DH5*α* and isolated using QIAprep spin miniprep kit (Qiagen). 293T cells were plated in 6-well plates 24 h before transfection. Cells were transfected with 2 *μ*g of pGL4.17 promoter FGF21 (−1497/+5) and 2 *μ*g of ATF4 or CHOP expression vector using Fugene HD (Promega). 48 h after transfection, the cells were harvested and lysed, and the luciferase activity was measured using the Luciferase Assay System (Promega). The transfection efficiency was normalized by cotransfection of 1 *μ*g of GFP vector.

### 3.6. Assessment of FGF21 mRNA Stability

3T3-L1 mature adipocytes were treated with TG (100 nM)/DTT or vehicle control for 4 h, followed by addition of actinomycin D (5.0 *μ*g/mL) to the medium (time 0). The total cellular RNA was isolated 0.5, 1, 2, 4, and 6 h after the addition of actinomycin D. The mRNA levels of FGF21, ATF4, XBP1sp, and CHOP were detected using real-time RT-PCR as described in the previous section and the results are expressed as the fold of the mRNA level at time 0.

### 3.7. Statistical Analysis

All of the experiments were repeated at least three times. Results are stated as the mean ± standard error. One-way ANOVA was employed to analyze the differences between sets of data. Statistics were performed using GraphPad Pro. A value of *P* < 0.05 was considered significant.

## 4. Results

### 4.1. ER Stress Increases FGF21 Expression

To investigate the effect of ER stress on FGF21 expression in adipocytes, 3T3-L1 adipocytes were treated with different concentrations of TG or for different time periods. The mRNA levels of ER stress-specific genes (ATF4, XBP1-sp, and CHOP) and FGF21 were detected using real-time RT-PCR. As shown in Figure 1(a), TG increased FGF21 mRNA expression in a time-dependent manner. However, the expression levels at 24 h were lower than that at 16 h, perhaps due to cell toxicity of TG. As shown in Figure 1(b), TG-induced FGF21 expression was concentration-dependent.

### 4.2. ER Stress Induces FGF21 Secretion

After a model of TG-induced stress in 3T3-L1 adipocytes was established, we further examined whether ER stress also increases FGF21 secretion. Differentiated 3T3-L1 cells were treated with TG and the FGF21 protein level in the medium was measured using ELISA. As shown in Figures 2(a) and 2(b), TG induced secretion of FGF21 in time- and dose-dependent manners. The FGF21 protein level in the medium was increased 40-fold after 24 h treatment with TG (100 nM).

### 4.3. Knockout of CHOP Decreases FGF21 Expression

CHOP is a major transcription factor involved in ER stress-induced apoptosis. To determine whether CHOP expression contributes to ER stress-induced upregulation of FGF21, we isolated MPH from WT and CHOP^−/−^ mice and treated the cells with TG for 24 h. In WT MPH, TG promoted the mRNA levels of CHOP and FGF21. However, in CHOP^−/−^ MPH, TG failed to induce FGF21 expression. Previous studies reported that ATF4 can induce FGF21 expression under stress conditions [33–37]. Since ATF4 is an upstream gene of CHOP, CHOP knockout had no effect on TG-induced activation of ATF4. As shown in Figure 3, with an absence of CHOP, TG-induced FGF21 was reduced by 30% compared to that in WT MPH. These results suggest that CHOP also plays a key role in TG-induced FGF21 expression in MPH.

### 4.4. ATF4 and CHOP Increase FGF21 Promoter Activity

To further identify the underlying mechanisms of ER stress-induced FGF21 expression, we subcloned the FGF21 full length promoter (−1497/+5) into the pGL4.17-Luc luciferase reporter vector and examined the effect of ATF4 and CHOP on regulation of FGF21 promoter activity by cotransfection of FGF21 luciferase reporter and ATF4 or CHOP overexpression vector. A previous study has reported that FGF21 expression could be promoted by overexpression of ATF4 [37]. Due to the low transfection efficiency of the 3T3-L1 cell line, HEK293 cells were used in this study. HEK293 cells were cotransfected with pGL4.17-FGF21 Luc luciferase reporter vector and the expression vector for ATF4 or CHOP. Luciferase activity was determined at 48 h after transfection. As shown in Figure 4(a), ATF4 overexpression enhanced FGF21 promoter activity more than 3-fold as compared to the control vector, which is consistent with the previously reported study [37]. Two conserved ATF4 binding sites in the promoter region of the FGF21 gene have been identified; ER stress-induced FGF21 expression can be mimicked by overexpression of ATF4. CHOP is one of the downstream transcription factors of ATF4. To determine whether CHOP is also involved in ER stress-induced activation of FGF21 transcription, we cotransfected HEK293 cells with the FGF21 promoter reporter construct and CHOP overexpression vector. As shown in Figure 4(b), similar to ATF4, CHOP overexpression significantly increased the FGF21 promoter activity. These findings indicate that both ATF4 and CHOP are involved in ER stress-induced activation of FGF21 transcription.

### 4.5. ER Stress Increases FGF21 mRNA Stability

Posttranscriptional regulation is a major mechanism for the expression of fast turnover cytokines and growth factors. To determine whether TG- or DTT- (dithiothreitol-) induced ER stress had any effect on FGF21 mRNA stability, we measured the half-life of FGF21 mRNA in TG/DTT-treated 3T3-L1 adipocytes. The results indicate that both TG and DTT significantly increase the half-life of the FGF21 mRNA, but have no effect on ATF4 mRNA stability (Figure 5).

## 5. Discussion

FGF21 acts as a hormone-like cytokine on multiple tissues to coordinate carbohydrate and lipid metabolism [4]. Clinical research has shown that serum FGF21 levels are higher in subjects who are overweight/obese and have NAFLD or type 2 diabetes [14–18, 20]. Similarly, circulating FGF21 concentrations in* db/db* mice were much higher than those in normal mice, as were the FGF21 mRNA levels in both the liver and white adipose tissue [6, 8, 22]. Previous studies have reported that FGF21 expression is upregulated by several transcriptional activators. For example, FGF21 is induced directly by PPAR*α* in response to starvation and ketotic states and by specific PPAR*α* agonists in the liver [23, 25]. In addition, in cultured adipocytes and adipose tissue, FGF21 expression is induced by PPAR*γ* [39–41]. The farnesoid X receptor (FXR) also increased FGF21 gene expression and secretion via direct binding to the FXR/retinoid X receptor binding site in 5′-flanking region of the FGF21 gene [42]. It also has been reported that glucose activation of carbohydrate response element binding protein (ChREBP) and retinoic acid receptor-related receptor *α* (ROR*α*) is involved in the upregulation of FGF21 mRNA expression in liver [43, 44]. PGC-1*α*-mediated reduction of FGF21 expression is dependent on the expression of its ligand, ALAS-1, and Rev-Erb*α* [45].

In addition, studies done by Frank G. Schaap suggest that FGF21 expression is regulated by ER stress [37]. The authors reported that FGF21 mRNA is increased by TG-induced ER stress in rat H4IIE cells and rat primary hepatocytes. Moreover, intraperitoneal injection of the ER stress activator, tunicamycin, induced hepatic FGF21 expression in mice and resulted in marked elevation of serum FGF21 levels [37]. Consistent with these new findings, we observed that TG-induced ER stress elevated FGF21 expression and secretion in murine 3T3-L1 adipocytes along with increasing ATF4 expression.

PERK (PKR-like ER kinase) is one of the major ER stress pathways. PERK can induce CHOP via activating ATF4. However, there was no information available regarding the regulation of FGF21 by CHOP. The current study showed, for the first time, that CHOP is also involved in regulating FGF21 expression at both the transcriptional and posttranscriptional levels under ER stress conditions. We analyzed the mouse FGF21 (−1497/+5) promoter and confirmed the absence of the conserved CHOP binding site, 5′-(A/G) (A/G) TGCAAT (A/C) CCC-3′, indicating CHOP may not directly regulate FGF21 transcription. However, our data demonstrates that CHOP significantly increased the FGF21 promoter activity (Figure 4(b)). CHOP may also regulate the expression of FGF21 indirectly by activating other cytokines and intracellular stress signaling pathways, though this remains to be further determined.

Gene expression can be regulated by posttranscriptional control of mRNA stability [46]. The presence of AU-rich elements (AREs) in the 3′-untranslated region (3′-UTR) is essential for stabilization or degradation of mRNAs involved in inflammatory and stress responses [47]. The RNA-binding proteins (RBPs), such as HuR, AUF1, and CUG-BP1, regulate the stability of many target mRNAs via binding to the AREs in the 3′-UTR [48, 49]. In this study, we identified for the first time that ER stress increased FGF21 mRNA stability in differentiated 3T3-L1 cells. However, the responsible RBPs remain to be further identified.

In conclusion, these findings suggest that FGF21 is a target gene of ATF4 and CHOP. Under ER stress conditions, ATF4 and CHOP induce FGF21 expression via activating promoter activity and stabilizing mRNA. Thus, our study indicates that activation of ER stress is the key event responsible for upregulation of FGF21 in several metabolic diseases. Moreover, our studies suggest that targeting ER stress-mediated signaling pathways represents a new therapeutic strategy for treatment of metabolic disease.

## Figures and Tables

**Figure 1:**
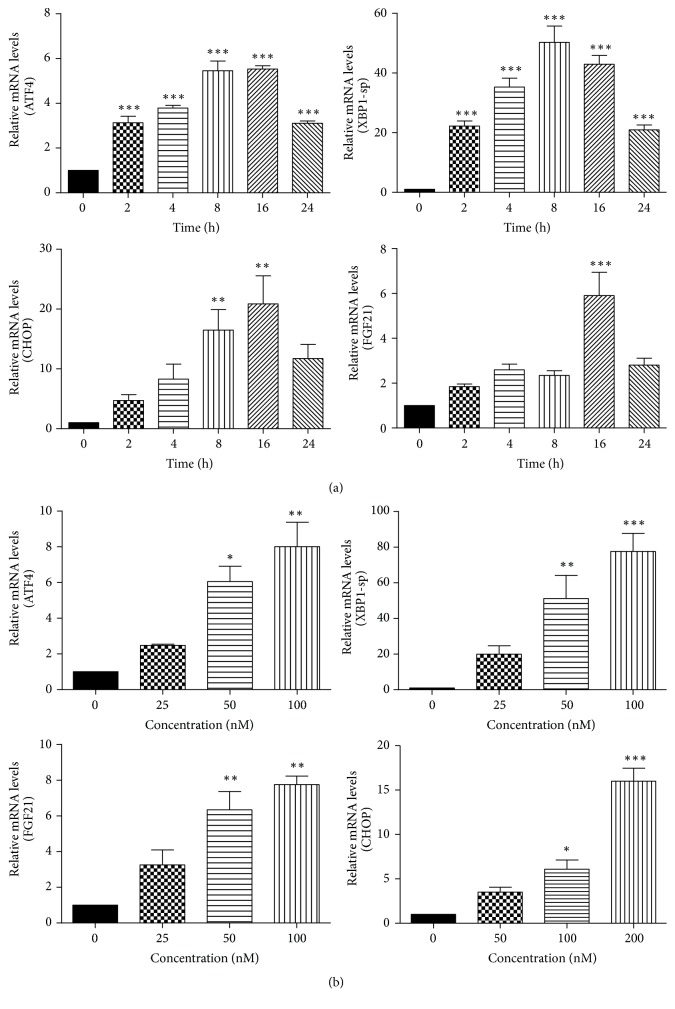
ER stress increases FGF21 mRNA levels. (a) 3T3-L1 adipocytes were treated with TG (100 nM) for 0, 2, 4, 8, 16, and 24 h; (b) 3T3-L1 adipocytes were treated with TG (25, 50, and 100 nM) for 16 h. Total cellular RNA was isolated. The mRNA levels of ATF4, XBP1-sp, CHOP, and FGF21 were measured by real-time RT-PCR. Values are mean ± SE of three independent experiments. Statistical significance relative to vehicle control: ^*∗*^ *P* < 0.05; ^*∗∗*^ *P* < 0.01; ^*∗∗∗*^ *P* < 0.001.

**Figure 2:**
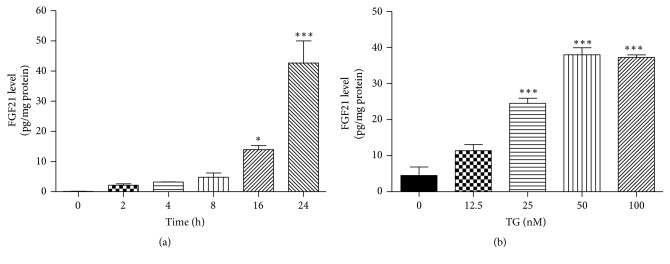
ER stress induces FGF21 secretion. Differentiated 3T3-L1 cells were treated with 100 nM TG for 0, 2, 4, 8, 16, and 24 h (a), or different concentrations of TG for 24 h (b); at the end of treatment, cell culture medium was collected. The protein level of FGF21 was determined by ELISA. Values are mean ± SE of three independent experiments. Statistical significance relative to vehicle control: ^*∗*^ *P* < 0.05; ^*∗∗∗*^ *P* < 0.001.

**Figure 3:**
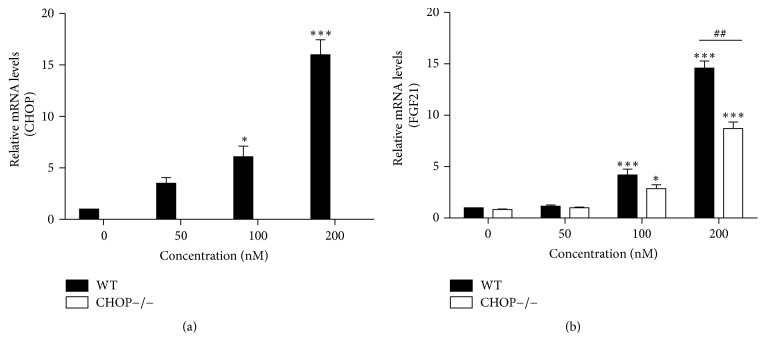
Knockout of CHOP decreases FGF21 expression. WT and CHOP−/− mouse primary hepatocytes were treated for 24 h with increasing concentration of TG. Total cellular RNA was isolated and the mRNA levels of CHOP and FGF21 were measured by real-time RT-PCR. Values are mean ± SE of three independent experiments. Statistical significance relative to WT vehicle control: ^*∗*^ *P* < 0.05; ^*∗∗∗*^ *P* < 0.001; statistical significance relative of the same TG concentration between WT group and CHOP−/− group: ^##^ *P* < 0.01.

**Figure 4:**
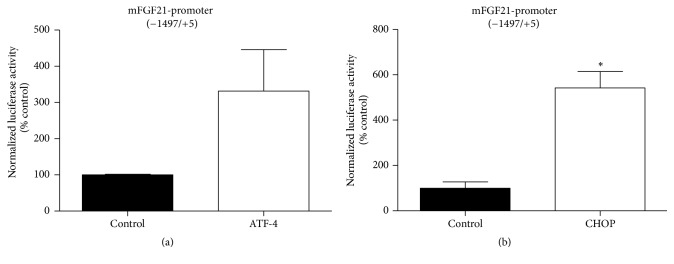
ATF4 and CHOP increase FGF21 promoter-driven transcription. 293T cells were transfected with FGF21 promoter reporter construct along with the expression plasmid ATF4 or CHOP. Values are mean ± SE of three independent experiments. Statistical significance relative to control vector: ^*∗*^ *P* < 0.05.

**Figure 5:**
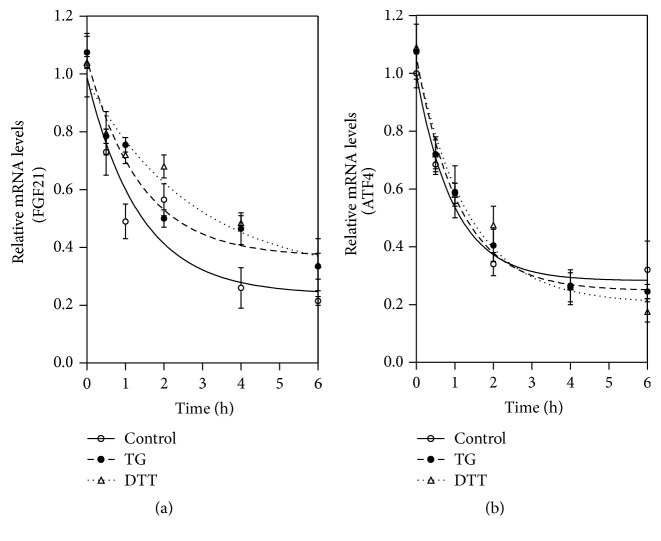
ER stress increases FGF21 mRNA stability. 3T3-L1 adipocytes were pretreated with 100 nM TG/DTT or vehicle control (DMSO) for 4 h and then treated with 5.0 *μ*g/mL actinomycin D (time 0). Total cellular RNA was extracted at 0, 0.5, 1, 2, 4, and 6 h after actinomycin D addition. FGF21 mRNA levels were determined by real-time RT-PCR. Values are mean ± SE of three independent experiments.
